# Association of postmenopausal endogenous sex hormones with global methylation level of leukocyte DNA among Japanese women

**DOI:** 10.1186/1471-2407-12-323

**Published:** 2012-07-29

**Authors:** Motoki Iwasaki, Hiroe Ono, Aya Kuchiba, Yoshio Kasuga, Shiro Yokoyama, Hiroshi Onuma, Hideki Nishimura, Ritsu Kusama, Teruhiko Yoshida, Shoichiro Tsugane

**Affiliations:** 1 Epidemiology and Prevention Division, Research Center for Cancer Prevention and Screening, National Cancer Center, 5-1-1 Tsukiji, Chuo-ku, Tokyo, 104-0045, Japan; 2 Division of Genetics, National Cancer Center Research Institute, 5-1-1 Tsukiji, Chuo-ku, Tokyo, 104-0045, Japan; 3 Biomedical Science PhD Program, Tokyo Medical and Dental University, 1-5-45 Yushima, Bunkyo-ku, Tokyo, 113-8510, Japan; 4 Department of Medical Oncology, Dana-Farber Cancer Institute, 450 Brookline Avenue, Jimmy-Fund JF-605, Boston, MA, 02215, USA; 5 Department of Surgery, Nagano Matsushiro General Hospital, 183 Matsushiro, Matsushiro-machi, Nagano-shi, Nagano, 381-1231, Japan; 6 Department of Breast and Thyroid Surgery, Nagano Red Cross Hospital, 5-22-1 Wakasato, Nagano-shi, Nagano, 380-8582, Japan; 7 Department of Surgery, Nagano Municipal Hospital, 1333-1 Tomitake, Nagano-shi, Nagano, 381-8551, Japan; 8 Department of Surgery, Nagano Hokushin General Hospital, 1-5-63 Nishi, Nakano-shi, Nagano, 383-8505, Japan

**Keywords:** Cross-sectional study, Endogenous sex hormones, Global methylation level, Luminometric methylation assay, Peripheral blood leukocyte DNA

## Abstract

**Background:**

Although global hypomethylation of leukocyte DNA has been associated with an increased risk of several sites of cancer, including breast cancer, determinants of global methylation level among healthy individuals remain largely unexplored. Here, we examined whether postmenopausal endogenous sex hormones were associated with the global methylation level of leukocyte DNA.

**Methods:**

A cross-sectional study was conducted using the control group of a breast cancer case–control study in Nagano, Japan. Subjects were postmenopausal women aged 55 years or over who provided blood samples. We measured global methylation level of peripheral blood leukocyte DNA by luminometric methylation assay; estradiol, estrone, androstenedione, dehydroepiandrosterone sulfate, testosterone and free testosterone by radioimmunoassay; bioavailable estradiol by the ammonium sulfate precipitation method; and sex-hormone binding globulin by immunoradiometric assay. A linear trend of association between methylation and hormone levels was evaluated by regression coefficients in a multivariable liner regression model. A total of 185 women were included in the analyses.

**Results:**

Mean global methylation level (standard deviation) was 70.3% (3.1) and range was from 60.3% to 79.2%. Global methylation level decreased 0.27% per quartile category for estradiol and 0.39% per quartile category for estrone while it increased 0.41% per quartile category for bioavailable estradiol. However, we found no statistically significant association of any sex hormone level measured in the present study with global methylation level of leukocyte DNA.

**Conclusions:**

Our findings suggest that endogenous sex hormones are not major determinants of the global methylation level of leukocyte DNA.

## Background

DNA methylation, which is one type of epigenetic change, may play an important role in carcinogenesis by silencing tumor suppressor genes through hypermethylation or by activating oncogenes through hypomethylation [1]. In addition to gene-specific DNA methylation, global DNA hypomethylation in regions that are normally methylated, such as repeats or transposable elements, can also lead to genomic instability and altered gene transcription, and thereby affect the normal growth and differentiation of target tissues [1,2]. Although the majority of methylation studies to date have focused on localized hypomethylation and hypermethylation of specific genes in tumors [3,4], several epidemiological studies have examined the global methylation level of peripheral blood DNA, which is considered a potential surrogate biomarker for systemic genome methylation status [5-9]. In consequence, global hypomethylation of leukocyte DNA has been associated with an increased risk of several sites of cancer, including breast cancer [5,6,8]. However, determinants of global methylation level among healthy individuals have remained largely unexplored, although a number of genetic and environmental factors are thought to influence the level of DNA methylation [10].

Given that global hypomethylation has been associated with an increased risk of breast cancer [8], as well as the important role of endogenous sex hormones in the etiology of breast cancer, particularly estrogens [11-13], we hypothesized that endogenous sex hormone level might be a determinant of the global methylation level of leukocyte DNA. Clarification of the association between sex hormones and global DNA methylation level may contribute to a better understanding of the etiology of breast cancer.

In the present study, we tested the hypothesis based on a cross-sectional study of postmenopausal Japanese women.

## Methods

### Study subjects

Subjects were postmenopausal women who participated as controls in a multicenter, hospital-based case–control study of breast cancer conducted from May 2001 to September 2005 at four hospitals in Nagano Prefecture, Japan. Details of this study have been described previously [14,15]. The study protocol was approved by the institutional review board of the National Cancer Center, Tokyo, Japan.

Briefly, the case subjects were a consecutive series of women aged 20–74 years with newly diagnosed, histologically confirmed invasive breast cancer who were admitted to one of the four hospitals during the survey period. Of the 412 eligible patients, 405 (98%) agreed to participate. Healthy controls were selected from medical checkup examinees at two of the hospitals and confirmed to not have any cancer. They were matched with one case subject each by age (within three years) and residential area during the study period. Among potential control women, one declined to participate and two refused to provide blood samples. Consequently, written informed consent was obtained from 405 matched pairs.

Of 405 control women, we selected postmenopausal women aged over 55 years who provided blood samples and reported an energy intake between 500 and 4000 kcal. The present study included a total of 185 women.

### Data collection

Participants were asked to complete a self-administered questionnaire which included questions on demographic characteristics, anthropometric factors, smoking habit, family history of cancer, physical activity, medical history, and menstrual and reproductive history. Dietary habits were investigated using a 136-item semi-quantitative food-frequency questionnaire (FFQ) which was developed and validated in a Japanese population [16,17]. In the FFQ, participants were questioned on how often they consumed individual food items (frequency of consumption), as well as relative sizes compared to standard portions. Daily food intake was calculated by multiplying frequency by standard portion and relative size for each food item in the FFQ. Daily intakes of nutrients were calculated using the fifth revised and enlarged edition of the Standard Tables of Food Composition in Japan [18]. The FFQ included questions on supplement use, but nutrient intake from supplements was not included in the analyses because no comprehensive database for supplements was available. Any influence is assumed to be minimal, however, because none of the present subjects used folate supplementation and only eight subjects (4.3%) used vitamin B supplementation. We previously documented that the questionnaire’s assessments of folate intake, and vitamin B2, B6, and B12 intake were reasonably valid [14,17].

Participants provided blood samples at the time they returned their self-administered questionnaire. Whole blood in 7-mL EDTA-2Na vacutainers and serum samples were stored at −80°C until analysis.

### Laboratory analysis

Details of hormone assays have been described previously [19]. Briefly, we measured estradiol, estrone, androstenedione, dehydroepiandrosterone sulfate (DHEAS), testosterone, and free testosterone in serum by radioimmunoassay. Bioavailable estradiol (free and albumin-bound estradiol) was measured by the ammonium sulfate precipitation method. Sex-hormone binding globulin (SHBG) was measured by immunoradiometric assay. Lower detection limits (LODs) were 5 pg/mL for estradiol, 15 pg/mL for estrone, 6.25 nmol/L for SHBG, 0.1 ng/mL for androstenedione, 5 ug/dL for DHEAS, 0.05 ng/mL for testosterone, and 0.4 pg/mL for free testosterone. Measurement values below the LOD were assigned half the value of the LOD if measurable values below the LOD were not available. Intra-assay coefficients of variation (CV) were 6.5% for estradiol at a mean concentration of 24.9 pg/mL (n = 12), 10.6% for bioavailable estradiol at a mean level of 48.1% (n = 10), 5.6% for estrone at a mean concentration of 101.7 pg/mL (n = 10), 4.7% for SHBG at a mean concentration of 104.6 nmol/L (n = 10), 9.4% for androstenedione at a mean concentration of 1.33 ng/mL (n = 10), 5.2% for DHEAS at mean concentration of 75 ug/dL (n = 10), 4.5% for testosterone at a mean concentration of 0.83 ng/mL (n = 10), and 11.6% for free testosterone at a mean concentration of 5.4 pg/mL (n = 10). A commercial laboratory performed all hormone assays and provided information on intra-assay CVs (Mitsubishi Kagaku Bio-Clinical Laboratories, Tokyo, Japan).

Genomic DNA was extracted from the peripheral blood using a Qiagen FlexiGene DNA Kit (Qiagen, Hilden, Germany) according to the manufacturer’s protocol.

Global DNA methylation was quantified by LUMA (LUminometric Methylation Assay) [20,21]. Three hundred nanograms of Genomic DNA were cleaved with HapII + EcoRI or MspI + EcoRI in two separate 20-μl reactions containing 2 μl of 10 × T buffer (330 mM Tris-acetate, 100 mM Mg-acetate, 660 mM K-acetate, 5 mM dithiothreitol), 2 μl of 0.1% BSA, and 5 units of each of the restriction enzymes. The reactions were set up in a PSQ 96 Plate Low (Qiagen) and incubated at 37°C for 1 h. Then, 20 μl of annealing buffer that contained 200 mM Tris-acetate and 50 mM Mg-acetate pH 7.6 was added to the cleavage reactions, and samples were assayed using PSQ96 MA system (Biotage AB, Uppsala, Sweden). The instrument was programmed to add dNTPs in six steps, including Step 1, dATPαS; Step 2, mixture of dGTP + dCTP; Step 3, dTTP; Step 4, mixture of dGTP + dCTP; Step 5, water; and Step 6, dATP. Peak heights were calculated using the PSQ96 MA software. HapII/EcoRI and MspI/EcoRI ratios were calculated as (dGTP + dCTP)/dATP for each reaction. The HapII/ MspI ratio was then calculated as (HapII/EcoRI)/(MspI/EcoRI), which corresponds to the proportion of unmethylated CCGG. Restriction enzymes (HapII, MspI, and EcoRI) were purchased from Takara Bio (1053A, 1150A and 1040A, respectively; Shiga, Japan). PyroMark Gold Q96 Reagents for pyrosequencing were purchased from Qiagen (972804). DNA quantification was performed using the Quan-iT PicoGreen dsDNA Reagent and kit (P7581, Invitrogen, CA, USA). Intra-assay CV was 6.4% at a mean level of 74% (n = 20).

Five polymorphisms in methylenetetrahydrofolate reductase (*MTHFR*) (rs1801133 and rs1801131), methionine synthase (*MTR*) (rs1805087), and methionine synthase reductase (*MTRR*) (rs10380 and rs162049) were genotyped by TaqMan SNP Genotyping Assays developed by Applied Biosystems (Foster City, CA). Genotype frequencies were tested for deviation from the Hardy–Weinberg equilibrium with the χ2-test as quality control for genotyping (all *P* values > 0.05).

### Statistical analysis

Hormone levels were divided into median or quartile categories. Adjusted mean global methylation levels of leukocyte DNA according to endogenous sex hormone levels were calculated using a multivariable liner regression model. To test linear trends for mean hormone levels, regression coefficients (β) were calculated in the multivariable linear regression model using median or quartile categories of individual hormone levels as ordinal variables. We performed the initial analyses with age-adjustment. In multivariate model, the following variables were used for adjustment: age, family history of breast cancer, history of benign breast disease, age at menarche, age at menopause, number of births, age at first birth, height, body mass index (BMI), smoking status, alcohol drinking habit, physical activity in the past five years, and energy-adjusted folate intake, most of which are known as potential breast cancer risk factors [11,12]. Dietary folate intake was adjusted for total energy intake with the use of the residual method [22,23]. To investigate potential effect modification, subgroup analyses were performed by dietary and genetic factors related to one-carbon metabolism and tests for interaction were carried out. All *P* values reported are two-sided, and significance level was set at *P* < 0.05. All statistical analyses were performed with SAS software version 9.1 (SAS Institute, Inc., Cary, NC).

## Results

Mean age (standard deviation [SD]) among the total of 185 women was 62.8 (5.7) years old and mean BMI (SD) was 23.4 (2.9) (Table 1). Measurement of global methylation levels of leukocyte DNA and endogenous sex hormone levels was completed in 185 women. Mean global methylation level (SD) was 70.3% (3.1) and range was from 60.3% to 79.2% (Table 2). The proportion of women with levels below the LOD were 1.6% for estradiol, 6.5% for estrone, 0% for bioavailable estradiol and SHBG, 0.5% for androstenedione and DHEAS, 41% for testosterone, and 95% for free testosterone. Because of the high percentage of women with levels below the LOD, we did not use free testosterone in the present analyses. Women with estrone values > 125 pg/mL, estradiol values > 75 pg/mL, or testosterone values > 125 ng/dL are suspected to use postmenopausal hormones but none of our present women met the above conditions.

**Table 1:** Characteristics of the study population

	**Mean**	**Standard deviation**
Age (years)	62.8	5.7
Height (cm)	152.9	5.5
Body mass index (kg/m^2^)	23.4	2.9
Age at menarche (years)	13.9	1.6
Age at menopause (years)	50.0	3.8
Age at first birth (years)^*^	26.2	3.3
	**Number**	**%**
Family history of breast cancer, n (%)	17	9.2
History of benign breast disease, n (%)	11	6.0
Nulliparous, n (%)	17	9.2
Number of births (4 or more births), n (%)^*^	6	3.6
Breast feeding (yes), n (%)^*^	154	93.3
Smoking (ever smoker), n (%)	6	3.3
Alcohol drinking (drinker), n (%)	67	36.2
Physical activity in past 5 years (yes), n (%)	85	46.5

^*^Among parous women only.

**Table 2:** Distribution of global methylation level in leukocyte DNA and endogenous sex hormone level among 185 Japanese women

**Mean**	**Standard deviation**	**Median**	**Inter-quartile range**	**Under lower detection limit**	
				**(number)**	**(%)**
Global methylation level in leukocyte DNA (%)					
70.3	3.1	70.2	(68.5, 72.3)	0	0
Estradiol (pg/mL)					
9.7	5.7	9.0	(7.7, 10.4)	3	1.6
Bioavailable estradiol (%)					
24.2	7.0	23.0	(18.7, 28.9)	0	0
Estrone (pg/mL)					
24.4	9.5	24.0	(18.0, 28.0)	12	6.5
Sex hormone binding globulin (nmol/L)					
80.4	35.4	76.3	(57.0, 97.4)	0	0
Androstenedione (ng/mL)					
0.73	0.31	0.70	(0.50, 1.00)	1	0.54
Dehydroepiandrosterone sulfate (DHEAS) (ug/dL)					
59.8	32.1	54.0	(38.0, 76.0)	1	0.54
Testosterone (ng/mL)					
0.07	0.08	0.07	(Not detected, 0.11)	76	41.1

**Figure 1:**
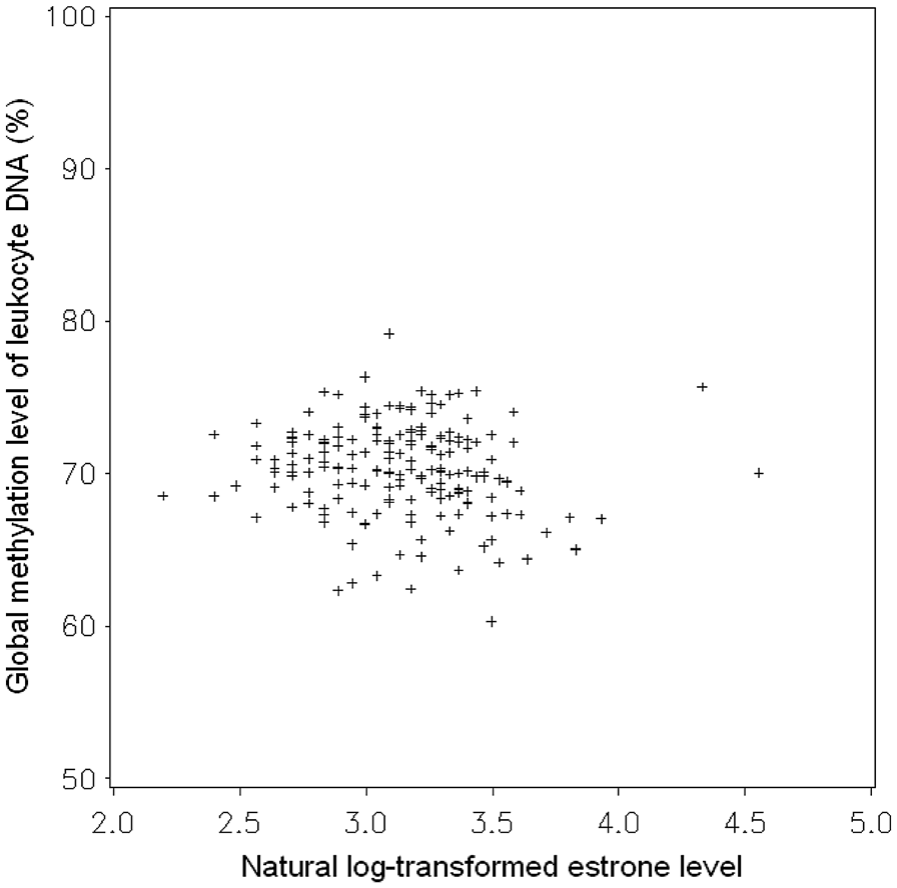
Scatter plot between natural log-transformed estrone level and global methylation levels of leukocyte DNA.

Mean global methylation levels according to endogenous sex hormone levels are shown in Table 3 and Figure 1. In the age-adjusted model, a higher level of estrone was significantly associated with a lower level of global methylation (0.45% decrease per quartile category, p = 0.03) but no statistically significant association was observed for other sex hormones (data not shown in Table). In the multivariate model we did not find a statistically significant association between any sex hormone level measured in the present study and the global methylation level of leukocyte DNA. However, higher levels of estradiol, estrone, androstenedione, DHEAS, and testosterone were associated with a lower level of global methylation while a higher bioavailable estradiol level was associated with a higher level of global methylation. Global methylation level decreased 0.27% per quartile category for estradiol (p = 0.20) and 0.39% per quartile category for estrone (p = 0.06) while it increased 0.41% per quartile category for bioavailable estradiol (p = 0.06). We further calculated regression coefficients per unit using a continuous variable of individual natural log-transformed hormone levels. The borderline statistically significant associations for estrone and bioavailable estradiol disappeared (p = 0.15 for estrone and p = 0.20 for bioavailable estradiol) and none of the associations were statistically significant (data not shown in Table).

**Table 3:** Mean global methylation level in leukocyte DNA by endogenous sex hormone level

	**Median**	**No. of subjects**	**Crude**	Multivariate-adjusted*				
			**Mean (%)**	**Mean (%)**	**95% confidence interval**	**β**	**95% confidence interval**	**P value**
Estradiol (pg/mL)								
Lowest	6.8	45	70.3	71.0	(68.9, 73.1)			
Second	8.2	45	70.8	71.5	(69.5, 73.4)			
Third	9.7	47	70.6	71.2	(69.3, 73.1)			
Highest	11.4	48	69.4	70.2	(68.2, 72.1)			
Continuous (per quartile category)						-0.27	(-0.68, 0.15)	0.20
Bioavailable estradiol (%)								
Lowest	16.6	45	69.5	69.7	(67.6, 71.8)			
Second	20.6	47	70.7	71.2	(69.3, 73.0)			
Third	26.0	44	70.5	70.8	(68.8, 72.9)			
Highest	32.5	49	70.4	71.2	(69.2, 73.2)			
Continuous (per quartile category)						0.41	(-0.01, 0.84)	0.06
Estrone (pg/mL)								
Lowest	15.0	38	70.6	71.6	(69.6, 73.7)			
Second	21.0	54	70.6	71.8	(69.8, 73.7)			
Third	25.5	40	70.8	71.8	(69.8, 73.9)			
Highest	31.0	53	69.3	70.4	(68.6, 72.3)			
Continuous (per quartile category)						-0.39	(-0.80, 0.02)	0.06
Sex hormone binding globulin (nmol/L)								
Lowest	44.1	46	69.9	70.9	(68.9, 72.8)			
Second	67.1	46	70.5	71.6	(69.6, 73.6)			
Third	84.9	45	70.8	71.7	(69.7, 73.7)			
Highest	113.5	48	69.9	70.7	(68.8, 72.6)			
Continuous (per quartile category)						-0.05	(-0.47, 0.37)	0.81
Androstenedione (ng/mL)								
Lowest	0.30	39	70.5	71.6	(69.5, 73.6)			
Second	0.60	44	70.1	71.2	(69.2, 73.1)			
Third	0.80	55	70.9	71.9	(69.9, 73.9)			
Highest	1.10	47	69.5	70.5	(68.7, 72.4)			
Continuous (per quartile category)						-0.24	(-0.67, 0.19)	0.27
Dehydroepiandrosterone sulfate (DHEAS) (ug/dL)								
Lowest	27.5	46	70.7	71.8	(69.7, 73.8)			
Second	45.0	45	69.8	70.7	(68.8, 72.7)			
Third	63.0	47	70.7	71.4	(69.5, 73.3)			
Highest	95.0	47	69.9	70.8	(68.8, 72.8)			
Continuous (per quartile category)						-0.24	(-0.66, 0.18)	0.26
Testosterone (ng/mL)								
Lowest	0.00	90	70.4	71.1	(69.3, 73.0)			
Highest	0.11	95	70.1	71.0	(69.1, 72.8)			
Continuous (per median category)						-0.17	(-1.10, 0.77)	0.73

* Adjusted for age (continuous), family history of breast cancer (yes, no), history of benign breast disease (yes, no), age at menarche (continuous), age at menopause (continuous), number of births (0, 1, 2-3, 4+), age at first birth (≤24, 25-26, ≥27, nulliparous), height (continuous), body mass index (continuous), smoking (never smokers, ever smokers), alcohol drinking (non-drinkers, occasional drinkers, regular drinkers), physical activity in past 5 years (no, ≤2 days/week, ≥3 days/week), and energy-adjusted folate intake (continuous).

We performed subgroup analyses of associations between sex hormone level and global methylation level by alcohol drinking status and folate intake (Table 4). A higher DHEAS level was significantly associated with a lower level of global methylation among alcohol-drinking women (0.78% decrease per quartile category), whereas no association was observed among non-drinkers (0.11% increase per quartile category) (*P* for interaction = 0.03). No statistically significant interaction was observed for other sex hormones, however. We further performed subgroup analyses by vitamin B2, B6, and B12, and five polymorphisms in *MTHFR, MTR,* and *MTRR* but found no statistically significant interaction (data not shown).

**Table 4:** Subgroup analyses of association between mean global methylation level in leukocyte DNA and endogenous sex hormone level by alcohol drinking status and folate intake

Multivariate-adjusted*			Multivariate-adjusted*			**P for interaction**
**β**	**95% confidence interval**	**P value**	**β**	**95% confidence interval**	**P value**	
**Alcohol drinking = Non-drinker**			**Alcohol drinking = Drinker†**			
Estradiol (pg/mL)						
-0.09	(-0.57, 0.39)	0.71	-0.52	(-1.37, 0.32)	0.22	0.16
Bioavailable estradiol (%)						
0.49	(0.01, 0.97)	0.04	0.26	(-0.60, 1.13)	0.54	0.09
Estrone (pg/mL)						
-0.28	(-0.77, 0.21)	0.26	-0.48	(-1.29, 0.33)	0.24	0.34
Sex hormone binding globulin (nmol/L)						
-0.11	(-0.61, 0.38)	0.65	0.03	(-0.77, 0.83)	0.95	0.26
Androstenedione (ng/mL)						
-0.23	(-0.74, 0.29)	0.39	-0.14	(-0.99, 0.70)	0.74	0.92
Dehydroepiandrosterone sulfate (DHEAS) (ug/dL)						
0.11	(-0.41, 0.63)	0.67	-0.78	(-1.56, 0.001)	0.05	0.03
Testosterone (ng/mL)						
-0.24	(-1.36, 0.88)	0.67	-0.20	(-1.95, 1.54)	0.81	0.44
**Folate intake = Low‡**			**Folate intake = High§**			
Estradiol (pg/mL)						
-0.05	(-0.71, 0.61)	0.88	-0.38	(-0.94, 0.18)	0.18	0.79
Bioavailable estradiol (%)						
0.64	(-0.02, 1.30)	0.06	0.26	(-0.37, 0.88)	0.42	0.47
Estrone (pg/mL)						
-0.30	(-0.92, 0.32)	0.34	-0.35	(-0.94, 0.24)	0.25	0.93
Sex hormone binding globulin (nmol/L)						
0.08	(-0.61, 0.77)	0.82	-0.12	(-0.73, 0.48)	0.69	0.74
Androstenedione (ng/mL)						
-0.67	(-1.36, 0.02)	0.06	-0.04	(-0.60, 0.51)	0.88	0.07
Dehydroepiandrosterone sulfate (DHEAS) (ug/dL)						
-0.45	(-1.07, 0.17)	0.15	-0.12	(-0.69, 0.46)	0.68	0.42
Testosterone (ng/mL)						
-0.82	(-2.30, 0.65)	0.27	0.45	(-0.86, 1.76)	0.49	0.08

* Adjusted for age (continuous), family history of breast cancer (yes, no), history of benign breast disease (yes, no), age at menarche (continuous), age at menopause (continuous), number of births (0, 1, 2-3, 4+), age at first birth (≤24, 25-26, ≥27, nulliparous), height (continuous), body mass index (continuous), smoking (never smokers, ever smokers), alcohol drinking (non-drinkers, occasional drinkers, regular drinkers), physical activity in past 5 years (no, ≤2 days/week, ≥3 days/week), and energy-adjusted folate intake (continuous).

† Median consumption of ethanol = 48.3 g per week.

‡ Median consumption of folate = 378.5 ug per day.

§ Median consumption of folate = 578.1 ug per day.

## Discussion

In this cross-sectional study among postmenopausal Japanese women, we found no statistically significant association between endogenous sex hormone level and the global methylation level of peripheral blood leukocyte DNA. To our knowledge, this is the first study to test the hypothesis that endogenous sex hormones are potential determinants of the global methylation level of leukocyte DNA. Our findings did not support this hypothesis, however, and suggest that endogenous sex hormones might not be major determinants.

Many epidemiological studies have confirmed the important role of endogenous sex hormones, particularly estrogens, in the etiology of breast cancer [11,12]. A pooled analysis of nine prospective studies showed that higher estrogens and their androgen precursors were associated with a higher risk of breast cancer in postmenopausal women [13]. In addition, several well-known risk factors have also implicated an etiologic role of sex hormones, including age at menarche and menopause, parity and age at first birth, and BMI [11]. Recently, Choi et al.. reported that global hypomethylation was associated with an increased risk of breast cancer [8], suggesting that the global methylation level of leukocyte DNA plays a role in the etiology of breast cancer. However, our findings suggest that global methylation level does not serve as a surrogate marker for the level of endogenous sex hormones and that the mechanisms by which global hypomethylation increases the risk of breast cancer might involve non-hormonal factors.

Several possible explanations for the observed absence of associations can be considered. First, misclassification due to inaccurate measurement may have attenuated the true association. However, since the reproducibility of assays for global methylation and sex hormone levels were relatively high, with an intra-assay CV for global methylation of 6.4%, and a range for hormones from 4.5% for testosterone to 10.6% for bioavailable estradiol, the absence of associations was unlikely due to measurement error. On the other hand, global methylation level might be affected by differences in the proportion of white blood cell types. Indeed, Zhu et al.. reported that the proportion of neutrophils and lymphocytes among white blood cell types modified LINE-1 methylation levels measured in blood DNA [9]. In the present study, since we measured global methylation level on white blood cells by the LUMA method, the possibility of measurement error due to different proportions of white blood cell types cannot be excluded. Such misclassification might accordingly have attenuated the true association, which might in turn have explained our results, at least in part. Second, the single time point measurement of serum hormone levels might not have captured the overall activity of the sex hormone-related signaling pathway of the subject up to the age of study. Third, although the present study included a total of 185 women, it may not have had sufficient statistical power to detect small associations.

Differences in assessment methods of global DNA methylation require consideration. While several previous studies estimated global DNA methylation based on data restricted to repetitive DNA elements, such as Alu and LINE-1 [7,8], the present study used LUMA, which allows the estimation of genome-wide DNA methylation levels. Differences in assessment methods might therefore have affected study results. For example, Choi et al.. found that global hypomethylation of leukocyte DNA measured by 5-methyldeoxycytosine (5-mdC) was associated with an increased risk of breast cancer but saw no association with the LINE-1 methylation level of leukocyte DNA [8]. Similarly, Wischwendter et al. reported no association between the Alu methylation level of leukocyte DNA and breast cancer risk [7].

The mechanisms by which endogenous sex hormone levels might affect global methylation status have not been elucidated. However, there is some evidence that estrogens or endocrine disruptors might alter DNA methylation status [24-26]. Studies in mice have shown that neonatal exposure to diethylstilbestrol (DES), a synthetic non-steroidal estrogen, induced hypomethylation of estrogen-responsive lactoferrin gene and proto-oncogene *c-fos* in utero [24,25]. Another study showed that exposure to persistent organic pollutants such as polychlorinated biphenyls, many of which are known endocrine disruptors, was associated with global DNA hypomethylation in Greenlandic Inuit [26].

In our subjects, subgroup analyses suggested that alcohol drinking modified the association between endogenous sex hormone levels and the global methylation level of leukocyte DNA: higher DHEAS was significantly associated with a lower level of global methylation among alcohol drinkers, whereas no association was observed among non-drinkers. Since alcohol consumption interferes with folate metabolism [27] and decreases levels of serum folate [28], our findings might indicate that higher sex hormone levels were associated with a lower level of global methylation among subgroups with a low folate status only. This hypothesis, however, does not appear to be supported by our subgroup analyses by folate intake or *MTHFR* polymorphisms (rs1801133 and rs1801131). Although the T allele of *MTHFR* (rs1801133) and C allele of *MTHFR* (rs1801131) have shown lower enzyme activity [29,30], no remarkable difference in global methylation level was observed among genotypes of *MTHFR*. These inconsistent findings might be explained by multiple comparisons, insufficient power, or the possibility that alcohol consumption interacts with sex hormone levels via a mechanism other than the folate-related metabolism. In any case, the findings of our subgroup analyses should be interpreted with caution, and further research to clarify effect modification by dietary and genetic factors related to one-carbon metabolism on the association between sex hormone level and global methylation level is clearly required.

## Conclusions

In this cross-sectional study among postmenopausal Japanese women, we found no statistically significant association between endogenous sex hormones and the global methylation level of leukocyte DNA. Although the interpretation should be limited to postmenopausal women, our findings suggest that endogenous sex hormones might not be major determinants of the global methylation level of leukocyte DNA and that mechanisms by which global hypomethylation increases the risk of breast cancer might involve non-hormonal factors. A comprehensive understanding of the determinants of the global methylation level of leukocyte DNA and the role of this level in the etiology of breast cancer awaits further study.

## Abbreviations

BMI, body mass index; CV, coefficient of variation; DES, diethylstilbestrol; DHEAS, dehydroepiandrosterone sulfate; FFQ, food frequency questionnaire; LOD, lower detection limit; LUMA, LUminometric Methylation Assay; MTHFR, methylenetetrahydrofolate reductase; MTR, methionine synthase; MTRR, methionine synthase reductase; SHBG, sex-hormone binding globulin.

## Competing interests

The authors declare that they have no competing interests.

## Authors’ contributions

All authors were involved with the study concept and design. MI, YK, SY, HO, HN, RK, and ST participated in the acquisition of data. HO and TY measured global DNA methylation level and genotyping. MI, HO, AK, TY, and ST contributed to the analysis and interpretation of data. MI and AK conducted the statistical analyses and MI wrote the manuscript. All authors participated in the interpretation of results and critical revision of the manuscript for important intellectual content. All authors read and approved the final manuscript.

## Pre-publication history

The pre-publication history for this paper can be accessed here:

http://www.biomedcentral.com/1471-2407/12/323/prepub
